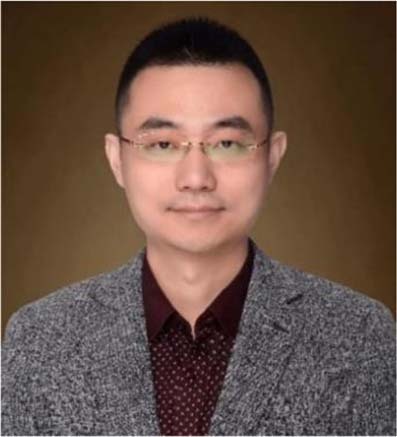# Light People: Professor Jianhua Jiang

**DOI:** 10.1038/s41377-021-00698-0

**Published:** 2022-01-05

**Authors:** Ying Zhang

**Affiliations:** Light Publishing Group, Changchun Institute of Optics, Fine Mechanics and Physics, Chinese Academy of Sciences, 3888 Dong Nan Hu Road, 130033 Changchun, China

## Abstract

Recently, Prof. Jianhua Jiang from Soochow University of China accepted an interview from *Light: Science & Applications*. Prof. Jiang works on topological photonics, topological phononics, and nonequilibrium physics. On this issue, he discusses the challenges and opportunities in topological photonics, topological phononics, and other topological synthetic systems. He also shares his experiences in cutting-edge research, the education of graduate students, and other challenges faced by junior researchers. Finally, he gives remarks and suggestions for *Light: Science & Applications*. Light People is a featured column of high-end interviews with outstanding scientists. It is our great honor to invite Prof. Jianhua Jiang, an outstanding young scientist, to showcase his research life and the story behind his success.

**Figure:**
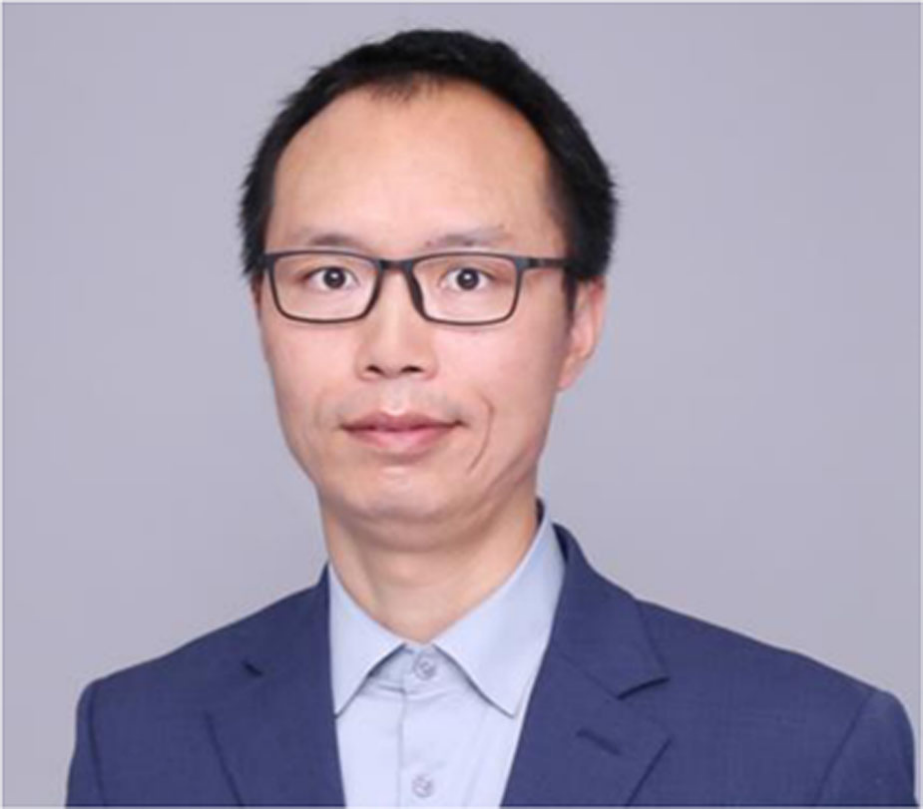

**Biography:** Prof. Jianhua Jiang graduated from the University of Science and Technology of China, where he obtained a bachelor’s degree in physics in 2004 and a Ph.D. degree in condensed matter physics in 2010. He later worked at the Weizmann Institute of Science in Israel and the University of Toronto in Canada as a postdoctoral fellow. He joined Soochow University in China in 2015 as a professor of physics. He worked on nonequilibrium physics and material physics for many years. In these research areas, he left his own marks with innovative and influential works that were published in *Nature, Nature Physics, Nature Materials, Nature Review Physics*, *Physics Reports*, and the *Physical Review* journals. He is a committee member of the National Conference on Statistical Physics and Complex Systems and of the National Conference on Metamaterials. He is also an associate editor of *Science Bulletin* and an editorial member of the *National Science Review*, *Chinese Physics Letter*, *Chinese Physics B*, and *Acta Phys. Sin*.

**Q1: Prof. Jiang, you pursued your graduate study at the University of Science and Technology of China and then performed postdoctoral research at the Weizmann Institute of Science in Israel and the University of Toronto in Canada. After this, you joined Soochow University in China as a professor. We know that you have worked in many different research directions in physics. Would you like to tell us what made you interested in the study of topological physics? How did your postdoctoral experiences affect your current research?**

When I was working at the Weizmann Institute as a postdoctoral fellow, at a certain time, it seemed that everyone was working on topological insulators. In such an environment, I started to learn topological physics and work on topological insulators. In a very short period, I completed several research projects^1–3^. One of them became the first work on Weyl semimetals in cold-atom systems^2^ and was recognized favorably by the community. However, my postdoctoral advisor, Prof. Yoseph Imry (2016 Wolf Prize in Physics laurate), was negative toward my working on such a popular research area. His reason was that, with the same amount of intelligence and energy, one could do much better and more recognized work in other fields. After carefully considering his opinions, I concluded that it is indeed very difficult to leave indelible marks in such a popular field (even though my work was later recognized by the top experts in the field). With this understanding, I quickly quit the field. Later, I realized that topological physics could be invaluable in photonics, phononics and related fields, and it could even lead to unprecedented applications, and I entered the field of topological physics again. However, this time, I focused on topological photonics and phononics. For this purpose, I went to the University of Toronto for another postdoctoral study with Prof. Sajeev John on photonic crystals and related physics. Prof. Sajeev John is a world-renowned expert on photonic crystals (In fact, he is one of the founding fathers of this field). My stay in Toronto was quite useful for me to learn photonics seriously and eagerly and to think more about how topological physics can be applied to photonics. On the other hand, my collaboration with Prof. Yoseph Imry never ceased. In fact, starting in 2012, we developed a new field called “inelastic thermoelectrics”. In this field, we study thermoelectric phenomena due to inelastic transport processes, which are distinct from conventional thermoelectric effects that are mainly due to elastic transport.

I like both fields, topological photonics/phononics and inelastic thermoelectrics. Therefore, when I started my group at Soochow University, I worked simultaneously in both of these fields. I mainly worked by myself in topological photonics/phononics and collaborated with experimental groups. However, my collaboration with Prof. Yoseph Imry continued and played an important role in my study of inelastic thermoelectrics. This collaboration was pleasant and fruitful until Prof. Yoseph Imry passed away in 2018. His influence on me is enormous. Most important were his taste and approaches in doing physics. He liked original and unique research. He was always pursuing originality and uniqueness in our studies. On the other hand, he was also a master of simplifying complicated projects, which was very helpful for me as a young researcher who was easily attracted by complex and “colorful” skills and techniques. His thinking on physics problems was a kind of distillation of the unique essence of the problems, and he put his energy toward developing small gems of new ideas. He did not like systematic studies or machinery tools. He just wanted a small gem of originality and uniqueness. It is in such very small gems that the essential beauty of science shines. In this sense, I learned science and research for the first time with Prof. Yoseph Imry. He showed me the remarkable value and beauty of simple things. From this whole process of training, I built my own taste of research as well as my confidence in all the research I have done because I can see the part of the value that would not fade away easily with time.

Meanwhile, Prof. Sajeev John also taught me many valuable things. The first thing I learned from him was how to put true values into research. He is very clear on the value of the studies we have done and knows how to present them properly. In my early years as a professor, I always asked his opinions on the value of my research and tried to get his feedback which are really helpful for me to clarify the valuable kernel of my research. My collaboration with Prof. Sajeev John continued after I left the University of Toronto. Prof. Sajeev John visited the Soochow University every year for our research collaborations and provided me the chance of visiting University of Toronto as well. His opinions and ideas have had a great influence on me and my colleagues. He substantially increased the quality and level of research on photonics at Soochow University.

**Q2: At the beginning of 2021, you and Prof. Yin Poo from Nanjing University in China made the experimental discovery of bulk-disclination correspondence in topological crystalline insulators published in** ***Nature***^4^**, which is an important milestone in the study of topological materials. In this work, photonic crystals are used as analogs of topological crystalline insulators to perform the experimental realization and study of disclinations at the macroscopic scale and the induced nontrivial topological effects. This opened a new pathway toward the experimental study of topological physics that was inaccessible before, giving fractional charges a central role in topological materials. What were the biggest challenges for you when working on this project? What are the opportunities and challenges that you could see for topological photonics in the future?**

The development of topological photonics and phononics has two driving motives. One is to utilize topological physics to discover new effects and phenomena in photonic and phononic systems or to improve the performance of photonic or phononic devices. The other is to exploit the advantages of photonic and phononic systems to realize and observe topological phenomena that are difficult to realize in condensed matter systems. In this regard, there are several advantages of photonic and phononic systems. First, photonic and phononic systems are highly tunable and can be fabricated with no difficulty. Second, the measurement methods in photonic and phononic systems are versatile. Moreover, these measurements are essentially out of equilibrium so that there is no limitation due to equilibrium distributions (e.g., band filling and temperature effects). Therefore, many topological phenomena can be observed in photonic and phononic systems at room temperature.

The famous work on the experimental observation of the quantum anomalous Hall effect by Prof. Qikun Xue and coworkers is well known in the community. One of the reasons is that it was an extremely difficult and challenging experiment. One challenge is that the bandgap of a quantum anomalous Hall system is very small. Therefore, the experimental system has to be in a clean state at a very low temperature. Otherwise, the disorder effect or the finite-temperature effect may ruin the quantized anomalous Hall conductance.

For photonic and phononic systems, because they are not restricted to equilibrium or near-equilibrium conditions, the experiments do not have to be performed at very low temperatures. For instance, even for GHz microwave photons, the photonic energy of which corresponds to 10 mK, the measurements can be done at room temperature because there are many more exciting photons than thermal photons in the experiments. In fact, one can perform photonic pump-probe measurements with a frequency resolution of 1 MHz or lower. Moreover, with the macroscopic realization of topological photonic systems, both the pump and the probe can be designed for various setups, allowing versatile measurements of the bulk and the boundary states.

In our recent papers^4,5^, we use topological photonic systems to realize an important principle: the band topology can be probed via defects. This is the long-sought bulk-defect correspondence^6^ that goes beyond the bulk-edge correspondence. Although it was proposed in 2009^6^ and later^7^, such fundamental phenomena were not confirmed until recently. In 2018, we demonstrated the first case of bulk-dislocation correspondence, where a dislocation in a magnetic photonic topological insulator leads to the formation of a robust defect mode^5^. This year, we used a disclination in an all-dielectric photonic topological insulator to verify the bulk-disclination correspondence^4^. The disclination not only leads to topological bound states but also leads to fractional charges. Such fractional charges bound to the disclination are sensitive to the crystalline symmetry, leading to fine experimental discernment of various topological crystalline insulator phases that cannot be distinguished via spectral properties such as bulk-boundary or higher-order bulk-boundary correspondences. Here, utilizing the nonequilibrium nature of photons, we are able to measure the photonic local density of states and hence reconstruct a fractional mode charge that has a one-to-one correspondence to the fractional charge in electronic topological insulators via band filling at zero temperature. I think this is a quite remarkable demonstration of the merits of photonic topological systems.

Currently, topological photonic crystals and other topological metamaterials have become one of the focuses of research on metamaterials. These materials provide new scopes for linear, nonlinear, and quantum optics. For instance, in nonlinear optics, topological lasers have demonstrated their advantages of greater stability and better performance. In addition, topological physics can be exploited to design stable and low-loss photonic crystal fibers. Topological modes bound to defects can be used as robust cavity modes. In the future, we expect more surprises from topological non-Hermitian, nonlinear, and quantum photonic systems.

**Q3: In 2019, you, Prof. Minghui Lu and Prof. Yanfeng Chen collaborated and achieved important progress on higher-order topological insulators published in** ***Nature Physics***^8^**. What is the main breakthrough in this work and your other works on higher-order topology? What do you expect from the future development of higher-order topological phases?**

I remember when we started to work on photonic topological systems with time-reversal symmetry, such as all-dielectric topological photonic crystals^9,10^. My group and other research groups are obsessed with gapless edge states, which are very difficult to realize in time-reversal symmetric systems since there is no Kramers degeneracy in photonics. I recall that in 2015, my students complained about the large edge bandgap in our photonic topological system. After that, I thought about how to make use of the edge gap instead of avoiding it, although the edge gap is commonly believed to be undesirable since it causes backscattering in the edge channels. At some time in 2016, I realized that this edge gap can be exploited to realize corner states via the Jackiw-Rebbi mechanism in bending edge channels. However, at that time, it was almost impossible for me to convince anyone to do the experiments. In 2017 and later, the concept of higher-order topological insulators became a popular idea in the study of topological insulators. I quickly realized that what we had found was in fact the higher-order topological insulator phenomenon. In collaboration with Prof. Lu and Prof. Chen at Nanjing University, we were able to quickly make important discoveries in higher-order topological insulators. Later, we continued to design and work together on various higher-order topological systems with gapped edge states and in-gap corner or hinge states.

In our 2019 work, we were among the first few teams to realize higher-order topological insulators for acoustic waves (the two other teams are from Nanyang Technological University in Singapore^11^ and the City University of New York in the USA^12^). The actual progress started in 2017 when we designed a new type of topological sonic crystal. In 2018, using this sonic crystal, we found the features of higher-order topological insulators: gapped edge states and in-gap corner states. Moreover, in our system, the topological transitions of the 2D bulk states and 1D edge states take place independently by tuning a single geometric parameter. That is, the edge state topological transitions can be triggered independently of the bulk state topological transitions. This feature is unique to our system and has never been observed before.

Later, we performed a series of innovative studies on higher-order topological phases^13–20^. In particular, we proposed the concept of higher-order Weyl semimetals^19,20^ and the symmetry-protected hierarchy of anomalous multipole topological bandgaps^14,17,18^. Because of our contributions to the field of higher-order topological phases, we were invited to write a review paper for *Nature Review Physics* on higher-order topology^21^. In this paper, research on various higher-order topological states was summarized and reviewed, and future perspectives and challenges were discussed. For instance, from an application perspective, corner states due to higher-order topology can be a good candidate for optical microcavity states and propagation modes in photonic crystal fibers. Looking back on all these points, I think we are truly lucky to have made innovative contributions to this field in the early stage. I believe that this had a lot to do with our difficulties back in 2016 and our determination to explore the puzzles we faced instead of avoiding them.

**Q4: Would you please tell us what difficulties and challenges you have ever encountered in your research? How did you deal with and overcome them?**

We, scientists, are trained to solve all kinds of problems and challenges starting in our student lives. We face different challenges in different stages of career development. At the beginning of my career, the main challenge was to find interesting research topics. In the next stage, the main challenge for me was how to define the value of my research on a long timescale. Now, my main challenge is to search for interesting and valuable research directions for my students, while I am keeping a thinking mind for doing valuable research. To address this challenge, both a long-term vision and a pragmatic approach are needed.

In recent years, I have always considered what the most valuable problems are that I can find and how I can focus energy and resources on them to create irreplaceable value. Although there are various difficulties when concrete ideas and strategies are carried out, the most difficult things are to form these ideas and strategies initially and to re-evaluate them from time to time.

**Figure:**
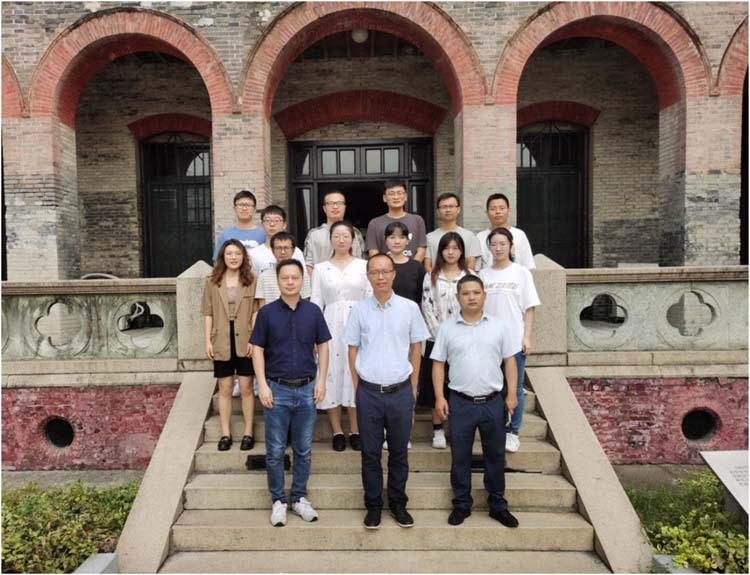
Prof. Jianhua Jiang’s group (photo taken at the 120-year-old building of Soochow University).

**Q5: Active young scientists are the assets of our community in both fundamental sciences and applications. The main challenges for them are probably applying for research funding and publishing high-quality papers. Could you please share your valuable experiences as one of the outstanding young scientists in your research field?**

Looking back at the decade after receiving my Ph.D. degree, I grew from technical maturity to thought maturity. In the first stage, I underwent various basic training (a continuation of Ph.D. training), including obtaining physics pictures, writing papers, giving talks, and collaborating with others. Then, I could conduct research independently and try to establish a research group and acquire, harness and retain more funding and resources. Just like other young scientists, I have been trying tirelessly to find a way to achieve success. If there is something special about me, it might be that I have high expectations and standards for my studies. That is, I don’t want to solve small problems or even those that many others are trying to solve. I keep thinking about what problems deserve to be solved and try hard to keep uniqueness and originality in my research. Of course, I have to combine strategies with practically doable things. I think leaving time to think, e.g., several years, before making a thing doable is important. During these years, another line of achievable research must be carried out.

To do great research, one has to have great visions and ideas. In any high-quality journal, the editors expect the publication to contain results that are the most innovative. This means that your ideas must go far ahead of most others. The formation of great ideas is something very important that has been missing in our daily research life and in the education of our graduate students. Many of our graduate students have narrow vision and little knowledge, but too much routine thinking. To build a strong community, we need to solve these problems. In recent years, in our community, we have probably also underestimated the importance of original research, especially for young scientists. Facing all these challenges, young scientists must form their own strategy and balance many things.

Work-life balance is also very important. I benefit from the support of a happy family. I have always believed that life is the foundation of work. I have received much support from my family and friends. I am really grateful for that.

**Figure:**
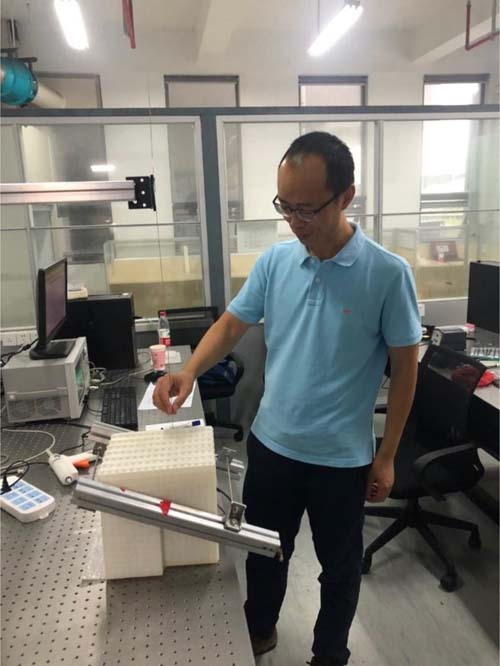
Prof. Jianhua Jiang in his laboratory.

**Q6: At the beginning of 2021, you published an important paper about topological photonics in** ***Nature***^4^**. Three of the four co-first authors are from the post-95 generation. Could you please share your experiences with training the students? Additionally, according to your experiences, could you please talk about how to create a good research environment and build a creative atmosphere in the research team?**

I am actually not sure that my experiences and strategies are valuable. Maybe I can share something. Often, at the beginning of Ph.D. training, I set low expectations for my students. However, I gradually increase it. In recent years, I have set my standards for selecting promising students. One of the first things that I consider is the motivation and quality of general education. These are very important qualities that may help graduate students get through the tough period of Ph.D. study. The other important quality I am looking for is the courage to walk outside of the sphere of one’s knowledge to do something in unknown territory. This is key to academic success. The students who are willing to try new things will be appreciated and fully supported by me. I will train them to try new things with some guiding principles. Once they get used to such training, they will grow up quickly. I believe that this will greatly benefit their confidence in research and have a long-lasting impact on their understanding of what constitutes scientific research. Education in innovation is invaluable for our research community. A good sign of a promising student is a thinking mind that is fearless and eager to explore the unknown.

Once the students are warmed up for innovative research, I will ask them to go out and give talks or to communicate with collaborators. I try not to ignore their training in other aspects outside of research. Communicating, giving talks, writing proposals, and solving problems in the lab are all very important for their academic success.

From my limited experience, I can tell that the young generation has much better prospects than my generation. They are more educated, informative, and creative. They are often more eager to communicate and have a better ability in communication. I expect them to do great things in the future.

**Figure:**
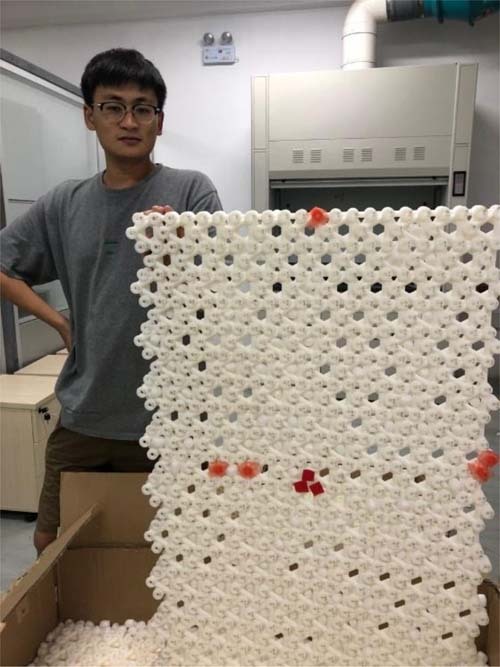
Mr. Zhikang Lin from Prof. Jiang’s group holds his experimental sample—a kagome acoustic metamaterial that hosts non-Abelian topological semimetal phases of phonons. (This work was published in *Nature Physics*^22^).

**Q7: In 2021, as an internationally-renowned journal in optics and photonics,** ***Light: Science & Applications*** **received the latest impact factor of 17,782, ranking the top 3 among all optics journals around the world for 7 consecutive years. Meanwhile, this journal won the China Publishing Government Award, which is the highest award in publishing in China. Looking back on nearly ten years of this journal’s founding history, 16 regions and overseas offices of** ***Light: Science & Applications*** **have been established at home and abroad. Series of brand activities based on** ***Light: Science & Applications*** **have been created and being emerging forward, such as Light Conference, Light Young Scientists Forum, Rising Stars of Light, Light Online, Light People, Light Academic League, Light Snapshot, etc. We also had the honor to invite you to attend the 2020 Light Nano-optics Young Scientists Forum held at Nanjing University in China, and you shared the report on fractional photonic charge in topological defects. Would you please talk about your feelings and experiences on** ***Light: Science & Applications*** **and its community activities?**

*Light: Science & Applications* has established a good reputation and its own development strategies. I believe that *Light: Science & Applications* will be a giant tree with prosperous developments one day. I think the development of *Light: Science & Applications* in the future would depend on its ability to build an ecological system around this tree. *Light: Science & Applications* has established its own brand. I believe that its future will be very impressive and it will have a long-lasting influence in the research community of optics and photonics, including basic, applied, and engineering research and applications.

**Figure:**
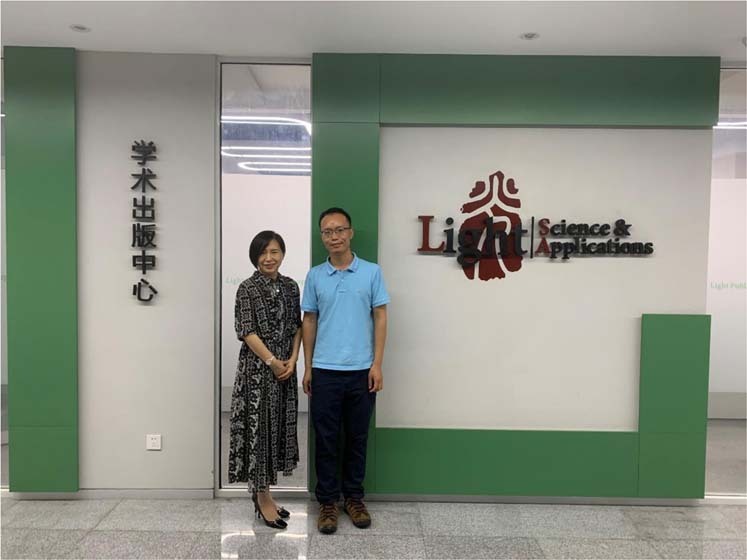
Photo with Prof. Yuhong Bai, the director of Light Publishing Group.

## Light correspondent

*Dr. Ying Zhang is the vice director of Light Publishing Group at Changchun Institute of Optics, Fine Mechanics and Physics (CIOMP), Chinese Academy of Sciences (CAS). He has been a visiting scholar at the Institute of Optics, University of Rochester during 2017–2018. He currently serves as the executive editor-in-chief of Chinese Journal of Liquid Crystals and Displays as well as an academic editor of Light: Science & Applications. He won the first prize and outstanding contribution award of project supported by STM Journal Society, CAS. He has hosted and participated in more than 10 provincial and ministerial projects of China. He has published over 40 SCI/EI and management papers. He also has presented over 30 invited talks and has been interviewed by China****’****s mainstream media such as the Xinhua News Agency. He participated in organizing and editing Handbook of Laser Technology and Applications (2nd Ed.) published by CRC, Taylor & Francis Group, as well as collection “Publishing Ethics of STM Periodicals” organized by China Association for Science and Technology*.

**Figure:**